# Application of Multiple Ultrasonic Techniques in the Diagnosis of Prostate Cancer

**DOI:** 10.3389/fonc.2022.905087

**Published:** 2022-06-27

**Authors:** Yushan Liu, Shi Zeng, Ran Xu

**Affiliations:** ^1^Department of Ultrasound, The Second Xiangya Hospital of Central South University, Changsha, China; ^2^Department of Urology, The Second Xiangya Hospital of Central South University, Changsha, China

**Keywords:** multiparameter ultrasound, prostate cancer, contrast - enhanced ultrasound, elastography, ultrasound molecular imaging

## Abstract

Methods for diagnosing prostate cancer (PCa) are developing in the direction of imaging. Advanced ultrasound examination modes include micro-Doppler, computerized-transrectal ultrasound, elastography, contrast-enhanced ultrasound and microultrasound. When two or more of these modes are used in PCa diagnosis, the combined technique is called multiparameter ultrasound (mp-US). Mp-US provides complementary information to multiparameter magnetic resonance imaging (mp-MRI) for diagnosing PCa. At present, no study has attempted to combine the characteristics of different ultrasound modes with advanced classification systems similar to the PIRADS system in mpMRI for the diagnosis of PCa. As an imaging method, mp-US has great potential in the diagnosis of PCa.

## Highlights

1. This article is a review of the application and development of various ultrasound techniques in the diagnosis of PCa.
2. Multiparameter ultrasound is a new combined mode of several ultrasound techniques, which is similar to multiparameter magnetic resonance imaging and it can significantly improve the diagnosis rate of PCa.
3. A complete ultrasound examination scoring system will have important clinical application value in improving PCa diagnosis and follow-up.

## Introduction

Prostate cancer (PCa) is the most common genitourinary system tumor in middle-aged and elderly men, and it is common in most Northern and Western countries. With the “Westernization” of lifestyles, the rapid aging of the population and the development of metabolic syndrome, the incidence and mortality of prostate cancer in our country have gradually increased in recent years (1, 2). The onset of PCa is insidious and lacks typical clinical manifestations. Most patients are already in the middle and late stages when they are diagnosed. Therefore, the early clinical diagnosis and treatment of PCa are of great significance in improving the survival rate of patients and their quality of life.

At present, early diagnostic tests of PCa mainly include on digital rectal examination (DRE), serum prostate specific antigen (PSA) measurement and conventional transrectal ultrasound (TRUS) (3). DRE is limited to palpation of the posterior area of the prostate, which can cause physical discomfort, rectal bleeding and even syncope. The level of PSA can indicate the risk of prostate cancer, but its sensitivity (SE) is high and specificity (SP) is low. The PSA of prostate cancer patients can even be in the normal range. Acute prostatitis and benign prostatic hypertrophy can also lead to an increase in PSA levels.Twelve-core systematic TRUS-guided biopsy for patients with serum PSA levels> 4.0 ng/mL is currently the gold standard for diagnosing PCa. Its SE is low, however, and the detection rate is only 27%-40.3% (4, 5). Additionally, the false negative rate of systemic prostate biopsy ranges from 17% to 21% (6, 7). Increasing the number of core biopsies can increase the detection rate of PCa and help better evaluate GS score (8, 9). The main disadvantage of systematic biopsy is that it is invasive, and can cause various complications such as prostatitis, hematuria, hematochezia, urinary retention and hematospermia (10). Additionally, it cannot detect small, low-risk, and clinically atypical cancers. Thus, it can lead to misdiagnosis, missed diagnosis, too many false negatives, and excessive puncture.

Therefore, an increasing number of researchers are dedicated to exploring imaging technologies with high SE, SP, and noninvasiveness. PCa imaging research focuses on two platforms: magnetic resonance imaging (MRI) and ultrasound (US). Multiparameter MRI (mp-MRI) is currently an important imaging method for PCa detection and localization and guidance of needle biopsy. The more commonly used sequences are T2-weighted imaging (T2WI), diffusion weighted imaging (DWI), dynamic contrast-enhanced MRI (DCE-MRI) and three-dimensional MR spectral imaging (11). However, MRI is not appropriate for claustrophobic patients, patients with pacemaker implantation and patients with metal pelvic implants. US is highly cost-effective and has wide applicability and strong practicability. Advanced US modalities include micro-Doppler, computerized-transrectal ultrasound, elastography, contrast-enhanced ultrasound and microultrasound. When different modes are used in combination, it is called multiparametric ultrasound (mp-US). This is a novel US examination mode similar to mpMRI, that can significantly improve the diagnosis rate of prostate cancer. This article introduces the basic principles and performance of different ultrasound-based modes and reports the clinical effects of combining them in mp-US.

## Greyscale TRUS

Currently, conventional TRUS is commonly used for prostate cancer detection, guided systematic biopsy, and guided radiotherapy particle placement (12). Because prostate cancer tissue and normal prostate tissue have similar backscatter signals and heterogeneity in the prostate transition zone, traditional TRUS has limitations in detecting PCa. Moreover, the higher frequency of the transrectal transducer can cause attenuation artifacts in the examination, especially when there are more calcifications in the prostate tissue. Approximately 60% of PCa lesions are hypoechoic on TRUS (13), and approximately 35–39% are isoechoic (14). Some nonmalignant diseases of the prostate, such as prostate inflammation and benign prostatic hyperplasia, can also appear hypoechoic on ultrasound images, leading to false positive test results.

The SE of TRUS in diagnosing PCa is between 8% and 88%, and the SP is between 42.5% and 99% (15–17). Taverna et al. observed that the PCa detection rate of 13-core TRUS-guided biopsy was 29% in 100 patients (18). A study by Klein et al. showed that TRUS has poor SP for early PCa, with a false negative rate for pathological results of systematic biopsy guided by TRUS of up to 30% (19). Hwang et al. noted that increasing the number of transrectal ultrasound-guided prostate punctures and the number of needles can increase the detection rate of PCa but would also increase the incidence of puncture complications (20). Therefore, targeted biopsy methods have arisen as the newest direction of research, as it can reduce the number of puncture needles and increase the detection rate of PCa. A study showed that the SP of TRUS-guided targeted biopsy in detecting PCa is better than that of mpMRI (41% vs. 96%) (21).

According to the European Urology Association (EAU) guidelines, standard grayscale TRUS remains the standard technique for biopsy guidance (22). The current US imaging system mostly uses nonlinear imaging. Its main advantage is the high contrast resolution of the tissue and low clutter in the inspection. The current trend in prostate ultrasound diagnosis is to increase the frequency of the probe and use broadband single-crystal piezoelectric elements to provide higher contrast and spatial resolution. In recent years, transrectal three-dimensional ultrasound (3D-TRUS) has been developed to provide more information for the diagnosis of PCa. Long et al. found that the accuracy and repeatability of needle biopsy guided by real-time three-dimensional ultrasound are better than those of two-dimensional ultrasound (23). Zhao et al. (24) and Guo et al. (25) showed that 3D-TRUS can help identify targeted puncture sites and increase the positive rate of PCa examination.

As a new high-resolution imaging method to guide prostate biopsy, microultrasound has received extensive clinical attention. Compared with mpMRI, microultrasound has potential advantages, such as relatively low cost and ease of operation. Several studies have reported the use of microultrasound in diagnosing PCa. In a meta-analysis of 769 patients, Zhang et al. found that microultrasound had a pooled SE, SP, diagnostic odds ratio (DOR), and area under curve (AUC) of 0.91, 0.49, 10, and 0.82, respectively (26). In 104 patients with suspected PCa, Lughezzani et al. found that the sensitivity and detection rate of microultrasound for the detection of csPCa were 94% and 54%, respectively (27). This study suggests that microUS can be used as an auxiliary diagnostic tool for MRI in diagnosis csPCa. PCa is a lower proportion of MRI-diagnosed PI-RADS 3 lesions, but prostate biopsy is still required. In a study of 111 patients, Pier Paolo Avolio et al. found that microUS detected 100% of csPCa in patients with a PI-RADS 3 lesion at mpMRI, while reducing the detection rate of ncsPCa to 23.8% (28). Sountoulides et al. (29) found that microultrasound-guided prostate biopsy has a PCa diagnosis rate equivalent to that of mpMRI-guided prostate biopsy. Laura Wiemer et al. found that the positive predictive value of micro-ultrasound for diagnosing csPCa was significantly higher than that of mpMRI in 159 patients (30). Microultrasound can be used as an inexpensive and convenient alternative to mpMRI in diagnosing PCa. Based on the findings of other studies, microultrasound can satisfactorily diagnose clinically significant prostate cancer (csPCa). The high SE of microultrasound in the diagnosis of PCa can further improve MRI/US-targeted biopsy and avoid unnecessary system biopsy. The diagnostic value of microultrasound still needs to be more comprehensively analyzed with more clinical data.

The latest EAU guideline (2022) recommend to perform, if possible, transperineal instead transrectal approach. In 200 patients with persistently elevated PSA, Pietro Pepe et al. found that transperineal MRI/TRUS cognitive targeted biopsy had a higher detection rate of csPCa in the anterior zone of prostate compared with transrectal MRI/TRUS fusion targeted biopsy (93.3 vs. 25%) (31). The transperineal approach provides relatively easy access to the anterior region, thus it reduces the patient’s risk of sepsis (32). In a study of 3000 patients with suspected PCa, Pietro Pepe et al. found that the detection rate of PCa by transperineal prostate biopsy was 38.3%, and 40.2% of the patients underwent biopsy without sepsis, only urinary tract infection and urinary retention (33). There are several studies focusing on fusion targeted biopsy and cognitive targeted biopsy, which one is more suitable for the detection of csPCa. In a study of 200 patients with persistently elevated PSA, Pietro Pepe et al. found that the sensitivity, specificity, positive predictive value, negative predictive value, and diagnostic accuracy of transperineal cognitive targeted biopsy in detection rate of csPCa were slightly higher than those of transrectal fusion targeted biopsy (97.2% vs. 66.7%, 78.2% vs. 71.8%, 59% vs. 42.1%, 97.2% vs. 87.5%, 68.9% vs. 57.5%, respectively.) (31). However, several studies have presented higher accuracy of MRI/TRUS fusion targeted biopsy compared with cognitive targeted biopsy, because the latter is operator-dependent (5, 34). In the past few years, mpMRI/TRUS fusion-targeted biopsy has improved the diagnostic accuracy of csPCa, especially in patients with repeat biopsies (35). More researches are still needed to present their respective advantages in diagnosing csPCa.

## Computer-Assisted Diagnostic System

In artificial neural network analysis/computerized-transrectal ultrasound (ANNA/C-TRUS), the doctor performs routine grayscale TRUS examinations on the patient before radical prostatectomy (RP). The images are sent to the ANNA/C-TRUS server through an internet platform. The C-TRUS system uses the ANNA algorithm to analyze the ultrasound images, then colors suspicious areas and returns them to the user terminal. This system is a convenient method for clinicians to performed guided, targeted tumor biopsy (36).

Among 132 patients with elevated PSA or abnormal DRE, 66 cases of cancer were found through C-TRUS targeted biopsy (37). Another study compared the tumor localization of the C-TRUS system before surgery and the pathological results after RP in 28 patients and found that the SE, SP, negative predictive value (NPV), positive predictive value (PPV) and total accuracy of the ANNA/C-TRUS system in detecting cancer were 83.1%, 63.9%, 68.4%, 80.1% and 76.2%, respectively (38). Moreover, the ANNA/C-TRUS system can better predict tumor differentiation than random systemic biopsy. In 164 preoperative patients undergoing RP, the SE of ANNA/C-TRUS in predicting the RP Gleason classification of the index lesions was 85% (39).When performing traditional TRUS, the additional use of C-TRUS can assist in the detection and biopsy of cancerous lesions. The combination of C-TRUS and MR can increase the detection rate for high-risk PCa patients (40). The use of ANNA/C-TRUS can improve the accuracy of PCa diagnosis, but a larger multicenter study is still needed to assess its clinical value.

Histoscanning (HS) is an ultrasound-based tissue characterization technology that can be used for PCa detection and localization. TRUS is used to first perform a full scan of the prostate to obtain three-dimensional grayscale data. Then the examiner uses HS software to color-code suspicious area and determine the tumor volume. This technique has shown encouraging results in the detection of csPCa.

In a study of 32 preoperative patients with RP, the SE, SP, PPV, and NPV of HS in detecting PCa were 93.5%, 79.5%, 67.35%, and 96.5%, respectively (41). HS can assist in diagnosing patients through prostate biopsy diagnosis. It has a higher detection rate for cancer lesions with a volume of ≥0.50 mL (42) and a diameter of ≥0.1cm (43). In 43 patients, the cancer detection rate of transrectal ultrasound biopsy with a standard 12-core system guided by prostate tissue scanning targeting (PHS-TT) was 46.5%, and the length of the PHS-TT cores was significantly higher than that of the systematic cores (55.4% vs. 37.5%, p <0.05) (44). PHS-TT can be used as an effective tool for the clinical guidance of prostate biopsy in real time.

In a study of 14 preoperative prostate HS in patients with RP, there was a significant correlation between tumor diameter and final pathology (r=0.95, p<0.001) (45). Simmons et al. observed a good correlation between tumor volume and final pathology (r=0.7) in a study of 27 patients, and the SE and SP of PHS in localizing of lesions ≥ 0.2 mL within a sextant were 90% and 72%, respectively (46). However, some studies arrived at different conclusions. A study of 148 PCa patients indicated that there was no significant correlation between the tumor volume measured by PHS and obtained in the final pathology (r = -0.0083, p = 0.9) (47). Javed S et al. also showed that the tumor volume measured by PHS was not correlated with the pathological volume after RP (r = -0.096) (48).

HS-targeted biopsy of the prostate is gradually being applied in clinical practice, but it still cannot replace the important role of systematic biopsy in detecting PCa. Compared with those of TRUS-guided prostate biopsy and transperineal template prostate biopsy (TTB), the overall cancer detection rates of PHS-targeted biopsy and TRUS-guided systemic biopsy are 38.1% and 62.5%, respectively (48). The total cancer detection rates of PHS-targeted biopsy and standard TTB were 13.4% and 54.4%, respectively (48). The SE and SP of PHS in the posterior gland were 100% and 13%, respectively, and those in the anterior gland were 6% and 82%, respectively (48). Therefore, it is currently not recommended to use HS to reliably identify and characterize PCa. The potential of PHS in assisting in the detection of PCa is considerable, and a larger patient population is still needed to further verify its clinical value.

## Color Doppler/Power Doppler

Several studies have reported the added value of Doppler technology over grayscale ultrasound (GSU) (9, 49, 50). Color Doppler ultrasound (CDU) and power Doppler ultrasound (PDU) can be used to detect invisible lesions on the GSU by revealing abnormal blood vessels in the tissue. CDU describes the speed and direction of blood flow by detecting the frequency changes when the signal is reflected by red blood cells (51). If the lesion is located in the peripheral zone of the prostate with nodular or clustered hypoechogencity, CDU manifests an intralesional vascular hyperplasia. Then, the lesion is likely to be malignant. Conventional CDU can improve the PCa detection rate (51).

PDU is another method of displaying blood flow in color, but it is more sensitive to perfusion than CDU. However, PDU cannot describe the direction of blood flow. PDU can detect low-velocity blood flow in blood vessels with an inner diameter as small as 1 mm. Okihara et al. used PDU to examine 107 men with high serum PSA levels. The results showed that the SE, SP, PPV and NPV of PDU in detecting of lesions were 98%, 78%, 59% and 99%, respectively (52). Sauvain et al. found that the SE and SP of PDU in detecting low-risk PCa in 243 patients were 45% and 74%, respectively (53). Eisenberg et al. compared GSU and PDU with 620 RP postoperative specimens and reported that adding PDU to GSU increased the SP from 47% to 74%, although the SE was reduced from 58% to 47% (15).

Both CDU and PDU can help identify vascular tissue, and the latter is more sensitive, but neither is sufficient to detect early PCa. Tumor growth and progression within the prostate are usually accompanied by angiogenesis, which may significantly increase the microvessel density (MVD) in the lesions. An increase in MVD is associated with a higher tumor grade and a worse prognosis (51). The limited resolution of Doppler ultrasound can detect blood vessels in the millimeter range, while the angiogenesis of malignant tumors can generate blood vessels as small as 10-50 microns in diameter (51). Therefore, the Doppler technique may be effective only in detecting increased blood flow in large vessels that are found in larger, advanced, high Gleason-grade tumors. Another potential disadvantage of Doppler and other blood flow-based ultrasound techniques is that the left-side lying position often used by patients may result in an asymmetrical distribution of blood flow in the prostate tissue. Harper et al. found that CDU and PDU showed a significant difference in blood flow in tissues (P<0.002) that are beneficial to the left side of the prostate instead of the right side (54).

## Elastography

Ultrasound elastography (UE) can reveal stiff lesions that are not visible on traditional TRUS (17, 55). The main methods for the UE diagnosis of PCa include transrectal real-time tissue elastography (TRTE) and shear-wave elastography (SWE). The index for evaluation with TRTE is the ratio of the stress on the material to the structural deformation caused by the stress, and the index for evaluation with SWE is expressed as the shear wave velocity and Young’s modulus.

### Transrectal Real-Time Tissue Elastography

In TRTE, the rectal probe cyclically compresses the suspicious prostate tissue and monitors the degree of elastic strain. The speckle comparison caused by each cycle of compression and decompression will generate a color-coded map, which is then overlaid on the grayscale image of the prostate. Note that the tissue deformation is homogeneous over the imaging plane, and the region of interest (ROI) should cover the entire gland and surrounding tissues to obtain a qualified and reproducible elastogram. Finally, the operator compares the tissue strain ratios of the two ROIs, with one considered “normal” and one considered “abnormal”, on the elastogram. On the elastic chart of the TRTE examination, low strain is highlighted by color coding in blue, and the corresponding high strain soft tissue is coded in red. Blue hypoechoic lesions of the prostate are suspected of malignancy. Normally, the stiffness of the glands in the prostate increases with age. PCa tissue is harder than normal prostate tissue due to increased cellular density, microvascularization and stromal reaction combined with collagen deposition in the surrounding prostate parenchyma (56). Thus, the organization of PCa tumors often involves partial or no obvious compression during TRTE inspection. The detection rate of prostate anterior parenchyma is lower than that in the posterior areas, and that of the base of the prostate is also lower than that of the apical regions in TRTE examination (55, 56).

Most studies on prostate elastography have used TRTE. A meta-analysis of 6 studies by Salomon G et al. showed that for TRTE targeted biopsy for PCa detection, the SE and SP per patient were 62% and 79%, respectively, and the SE and SP per core were 34% and 93%, respectively (57). Zhang B et al. compared TRTE with histopathological results after RP in a meta-analysis of 508 patients, and the pooled SE and SP of TRTE in diagnosing PCa were 0.72 and 0.76, respectively (58). Miyanaga et al. analyzed 29 patients with PCa before RP. The results showed that the SE of TRTE, GSU, and DRE in diagnosing PCa were 93%, 59%, and 55%, respectively (59).

Aigner et al. reported that in 94 patients, the SE, SP, PPV, and NPV of TRTE targeted biopsy were 74.0%, 60.0%, 39.0%, and 93.0%, respectively. Furthermore, TRTE-targeted biopsy was better than systemic biopsy, and the detection rate of PCa was 4.7 times higher (60). A comparative study of TRTE targeted guided needle biopsy and systemic biopsy by Brock et al. showed that TRTE had a higher positive rate for prostate needle biopsy than TRUS, but TRTE targeted guided needle biopsy was still unable to replace systemic needle biopsy (55). Therefore, we believe that TRTE-guided targeted biopsy can complement traditional systematic biopsy.

A study of 33 patients showed that the PCa detection rate of TRTE is basically equivalent to that of MRI. The SE and NPV of TRTE were 84.6% and 86.7%, respectively, while those of mpMRI were 84.6% and 83.3%, respectively (61). Pelzer et al. found that the SE and SP of TRTE in diagnosing PCa in 46 patients were 44.1–58.9% and 83.0–74.8%, respectively, while those of MRI were 36.7–43.1% and 85.9–79.8%, respectively (62). TRTE has advantages in the apical and middle parts of the prostate, while MRI has advantages in the gland base and TZ. The combination of the two detection methods can increase the total PCa detection rate (62). A study involving 41 patients showed that lesions on ventral prostate sectors were easier to detect by MRI, while TRTE more easily detected lesions in dorsal and apical sectors. The combination of MRI-TRTE significantly increased the area under the mpMRI curve from 0.65 to 0.75 (63). Brock et al. found that the SE and SP of the combined MRI/TRTE in detecting PCa were 77.8% and 77.3%, respectively (64).

Among the limitations of TRTE are that it performs a semiquantitative analysis of tissue elasticity; it cannot provide uniform compression for the whole gland; it has a low detection rate for small and low-grade prostates (65); and insufficient image acquisition and low reproducibility of the operation when the probe slips off the prostate, as shown for 32% of patients (66). Real-time balloon inflatable elastography (RBIE) has been adopted by clinics as a new technology for supplementing TRTE. It uses a pistol syringe connected to the balloon on the rectal probe to apply force to the prostate through inflation and deflation instead of manual compression. RBIE can more sensitively detect tumors with higher Gleason scores and hard-to-reach tumors in the prostate area. RBIE provides stable elastic motion images and improves the ability of TRTE to detect prostate cancer (67).

### Shear–Wave Elastography

In recent years, SWE has been primarily used for the diagnosis of thyroid, breast and liver diseases. SWE evaluates the hardness of the tissue by measuring the propagation speed of a shear wave delivered to the tissue. It is a quantitative technique that standardizes the detection of prostate cancer. The SWE measurements have excellent in-observer repeatability (ICC = 0.876) (68). However, SWE is plane-dependent, and the hardness of the sagittal image of the prostate is higher than that of the axial image (69); the shear waves attenuate significantly in larger glands; and for larger prostates, it is difficult to perform SWE without prepressurization.

What distinguishes SWE from TRTE is that the former avoids putting pressure on the rectal wall. The color rendering mode of SWE is opposite that of TRTE; low strain is highlighted in red, and soft tissues are shown in blue. Red hypoechoic areas are suspicious of malignant lesions. In young men without prostate hyperplasia, the area around and in the center of the prostate is uniformly displayed in blue, and the stiffness value ranges from 15 to 25 kPa. As prostate hyperplasia develops, the central area of the prostate becomes an uneven red with stiffness values ranging from 30 to 180 kPa, while the surrounding area still maintains a more uniform blue color (70). While attempting not to compress the prostate during SWE examination, the prostate is scanned from base to apex to obtain the original elastic image containing each plane. Then, the operator calculates the elasticity measure (mean, min and max) of each ROI, as well as the ratio between the quantitative box (Q-box) placed in the suspicious prostate area and the adjacent normal surrounding area.

SWE is a commonly used ultrasound imaging method for PCa diagnosis in the clinic and shows good diagnostic value. In a prospective study of 53 patients, a Young’s modulus value of 37 kPa was used as the cutoff value between benign and malignant prostate tissues. The SE, SP, PPV and NPV of SWE in detecting PCa were 96.2%, 96.2%, 69.4% and 99.6%, respectively (71). The meta-analysis results of Sang et al. showed that the pooled SE and SP of SWE in diagnosing PCa were 0.844 and 0.860, respectively (72). Boehm K et al. used 50 kPa as the Young’s modulus threshold for benign and malignant prostate tissues, and the SE and SP of SWE in detecting PCa were 80.9% and 69.1%, respectively (70). At present, the results of some studies using SWE show that the critical value for distinguishing benign and malignant lesions is in the range of 35 to 43.9 kPa (71, 73, 74).

The increase in PCa tissue stiffness is related to the GS (75) and disease severity (76). The average Young’s modulus of prostate cores with a Gleason score of 7 (163 ± 63 kPa) was higher than that of prostate cores with a Gleason score of 6 (95 ± 28.5 kPa; P = 0.007) (77). Woo et al. reported that Young’s modulus was significantly correlated with the Gleason score (r = 0.343, P = 0.002) (r = 0.898, P <0.0001) (73, 78, 79). Similarly, there is a correlation between the strain index (SD) and the Gleason score. The mean elastic strain index SD (3.26~1.77) of malignant focal lesions was found to be significantly higher than that of benign focal lesions (2.16~1.52; P<0.008), and the strain index was moderately linearly correlated with the Gleason score (r=0.441; P=0.013) (55). This finding may be attributed to the higher cell density and stiffness associated with higher grades of prostate cancer.

Rui et al. reported a new 11-point scoring system based on SWE and other clinical parameters (TRUS, DRE, and free PSA/total PSA ratio), and the results showed that when scoring based on SWE and clinical parameters, the AUC of the system (0.911) was higher than that of SWE alone (0.842) or of clinical parameters (0.868) alone (80). Recently, research has been conducted on the efficacy of 3D SWE in detecting prostate cancer. When the critical value of tissue elasticity of 41 kPa was combined with the PI-RADS score, the SE, SP, PPV and NPV of cancer detection were 70%, 98%, 91% and 92%, respectively (79). In the future, 3D SWE may have the potential to improve the detection of major prostate cancer.

### Acoustic Radiation Force Pulse Imaging

Acoustic radiation force pulse imaging (ARFI), another mode of UE, shows promise in the diagnosis and treatment of PCa. In ARFI, a short-term high-intensity focused ultrasound beam is transmitted to the prostate tissue to displace it. Zhai et al. successfully distinguished benign hyperplastic nodules, calcifications and cancerous lesions in the prostate using ARFI imaging (81). Wang et al. noted that a high-intensity ultrasound pulse can separate prostate cancer tissue from normal tissue, a potential noninvasive prostate cancer resection technique that and has therapeutic value (82).

## Contrast−Enhanced Ultrasound

A large number of microvessels are generated inside PCa tumors, which provide the necessary nutrients for tumor proliferation, metastasis and invasion. The density of microvessels in a PCa tumor is significantly higher than that of normal prostate tissue. In contrast-enhanced ultrasound (CEUS), an intravenous injection of ultrasound contrast agent (UCA) with a diameter close to red blood cells is made to observe the blood perfusion of the lesion and adjacent tissues in real time. CEUS can detect blood flow signals in microvessels with a diameter of 1–10 mm (83). The main component of the UCA is microbubbles (MBs), the incidence of allergic reactions is much lower than that of iodine contrast agents (84), and there is no nephrotoxicity. After intravenous injection of the UCA, one ROI is delineated in the suspicious area, and another is drawn in the enhanced normal parenchyma as a reference. The signal intensity change of the contrast agent in the prostate ROI area is plotted over time, which is called the time intensity curve (TIC). PCa tissue shows higher peak enhancement, and a shorter rise time and peak time than normal parenchyma (85).

In a prospective study of 65 patients with elevated PSA, Zhao et al. found that the SE and SP of CEUS in diagnosing PCa were 79.3% and 86.1%, respectively (86). In a meta-analysis of 16 studies with a total of 2624 patients, Li et al. found that the SE, SP, and DOR of CEUS imaging in detecting prostate cancer were 0.70, 0.74 and 9.09, respectively (87). Sedelaar et al. performed three-dimensional contrast-enhanced Doppler ultrasound (3D CE-PDU) on 7 patients with PCa confirmed by biopsy and found that the MVD on the “enhanced” side was 1.93 times that on the “unenhanced” side (88). Using 3D CE-PDU, 86% of cancer foci were found in 70 patients with PCa who planned to undergo RP (89). 3D CE-PDU has the ability to visualize lesions with high MVD.

CEUS-guided prostate targeted biopsy is widely used in clinical PCa detection. In a study of 1,776 men, Mitterberger et al. found that the PCa detection rate of CEUS–targeted biopsy was significantly higher than that of systematic biopsy (10.8% vs. 5.1%) (90). In a study involving 690 patients, Strazdina et al. found that CEUS–guided targeted biopsy had good SE in the detection of PCa with high Gleason scores (6 or higher) (91). Some studies have shown that targeted needle biopsy guided by CE-TRUS can not only improve the diagnostic SE of PCa but also increase the positive rate of needle biopsy (92–94). However, several studies have instead shown that there is no significant difference in the detection rate of PCa between the CEUS guided targeted puncture method and the systematic puncture method (95). CEUS is a promising tool for detecting PCa, but it still cannot completely replace systematic biopsy under existing circumstances.

Compared with other ultrasound modes, CEUS shows good diagnostic value in the diagnosis of PCa. Among 115 men with a serum PSA level greater than 4.0 ng/ml, a study showed that the SE, SP and accuracy of CEUS in diagnosing PCa were 65%, 83% and 73%, respectively, which were higher than those of TRUS and PDU (96). However, Taverna et al. reported that CEUS did not significantly increase the detection rate of PCa over PDU or GSU (18). Some recent studies used CEUS in combination with other ultrasound modes to detect PCa. Halpern et al. and Matsumoto et al. found that the total SE of CEUS and GSU in the diagnosis of PCa in 12 and 50 prostate cancer patients was 42% and 40%, respectively (97, 98). The combination of multiple ultrasound modes can significantly improve the ability to detect PCa clinically.

Contrast-enhanced ultrasound diffusion imaging (CUDI) is a very promising new technique for prostate cancer imaging developed in recent years. It analyzes the time evolution of the UCA concentration in the neovasculature of cancer foci to generate quantitative maps of perfusion parameters to better characterize microvascular structure. Jung et al. measured ultrasound contrast perfusion quantitative parameters in 20 PCa patients, including the early irrigation rate (EIR), mean transit time (MTT) and rise time (RT). The results showed that the SE, SP, NPV and PPV of PCa were 88%, 100%, 60% and 90%, respectively (99). This preliminary study shows that the quantitative analysis of CEUS perfusion parameters can help visualize the microvascular blood circulation and preoperative location of prostate cancer. In a study of 82 patients, Francesco M. Drudi et al. found that the sensitivity of mpMRI and quantitative analysis of contrast-enhanced ultrasound (CEUS) for detecting PCa were 91.3% and 40%, respectively, and the specificity were 66.7% and 97.2%, respectively (100). CUDI has also been studied in three dimensions. In a study using 3D CUDI to detect the PCa tumors in 43 patients, perfusion parameters were significantly different between benign and malignant tissues, including correlation (r) and wash-in time (WIT). The SE and SP of r in detecting PCa were 94% and 50%, and those of WIT were 53% and 81% (101).

Ultrasound molecular imaging is a new direction in the field of the early diagnosis of tumors. Due to the size limitation of MBs, CEUS is limited to the vasculature where MBs accumulate in the tumor. Only particles with a diameter of less than 700 nm can penetrate the tumor blood vessel wall and enter the tumor interstitium (102). Prostate-specific membrane antigen (PSMA) is a type II glycoprotein that is mainly distributed in prostate epithelial cells. It is highly expressed in prostate intraepithelial neoplasia, hormone-dependent or hormone-independent prostate cancer, and metastatic cancer (103) but expressed at low levels in normal prostate epithelial cells. This feature makes it one of the most important biomarkers in the diagnosis and treatment of PCa. Therefore, some studies focused on the construction of targeted nanobubbles (NBs) with a diameter of less than 700 nm to achieve specific ultrasound-enhanced imaging of prostate cancer cells (102–104). At present, PSMA-targeting, indocyanine green (ICG)-loaded nanobubbles (NBs) (102) and PSMA single-chain variable fragment (scFv)–loaded NBs have been reported (104). These new targeted NBs have been proven to be excellent US contrast agents that extend the signal enhancement time and have stronger penetrating ability and higher specificity (105, 106). If the NBs are loaded with drugs, targeted therapy of PCa can also be achieved.

## MRI/US Fusion Imaging

**Figure 1:**
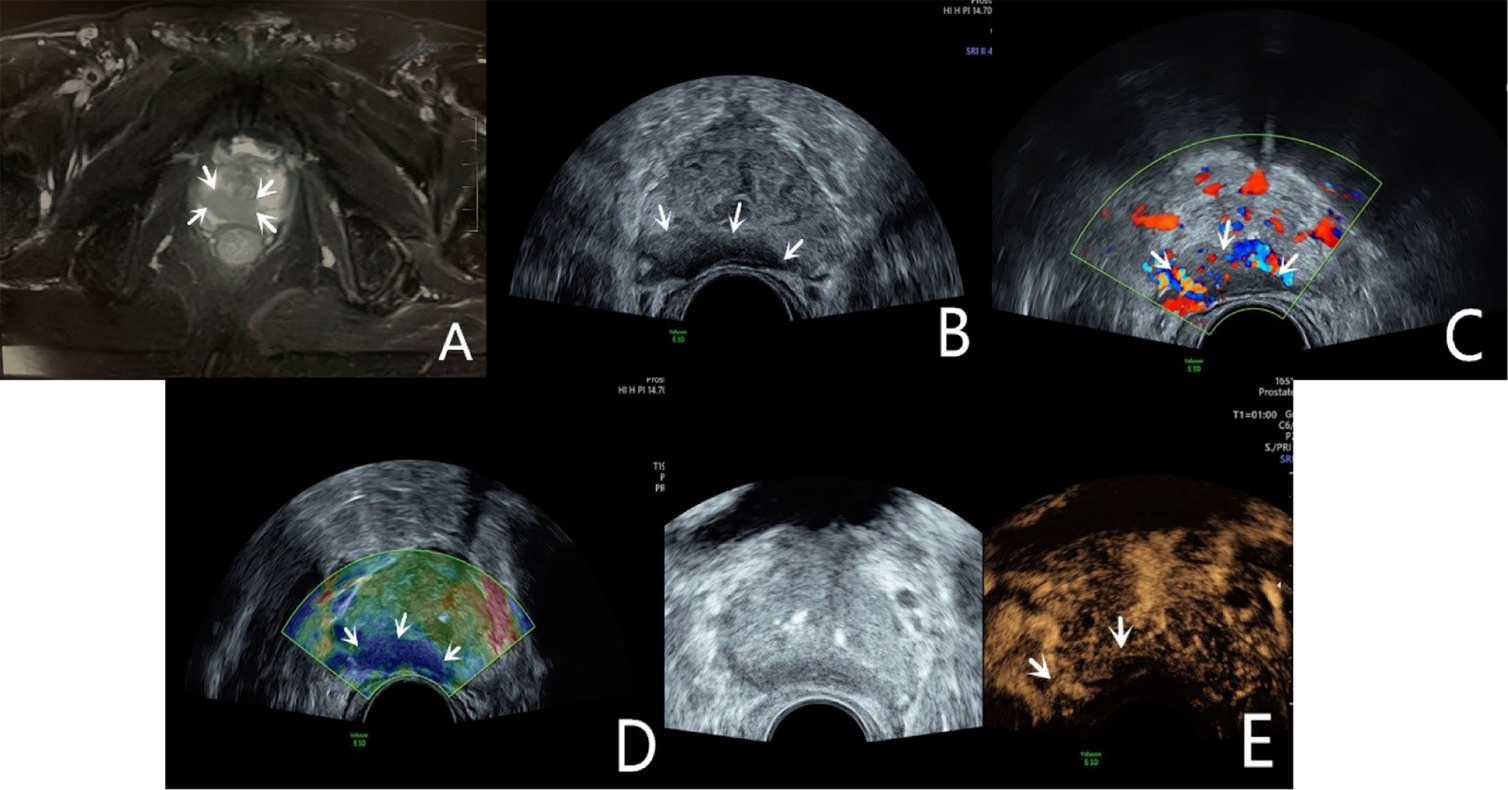
Diagnosis of PCa using mpUS—case 1. A 68-year-old patient has a total serum PSA of 10.4ng/ml. The T2-weighted sequence of MRI (**A**, arrow) shows a slightly low signal shadow in the peripheral zone of the prostate, suggesting PCa in the diagnosis. The lesion showed a slightly hypoechoic area on the B-mode (**B**, arrow), and it’s not clearly demarcated from the seminal vesicle gland. CDU shows an abnormally increased blood flow in the lesion (**C**, arrow). TRTE shows that the slightly hypoechoic area of the prostate’s peripheral zone is highly stiff (**D**, arrow). CEUS shows early high enhancement within the lesion (**E**, arrow). TRUS-guided systematic biopsy confirmed that the peripheral zone of the prostate was a Gleason 4 + 4 PCa.

When a lesion is detected on MRI, MRI/US fusion can be helpful (**Figure 1**). A number of studies have demonstrated that MRI/US fusion technology-guided biopsy improves the detection rate of PCa. Brock et al. found that using MRI/TRUS fusion targeted biopsy in 121 men, the SE and SP in the detection of PCa were 77.8% and 77.3%, respectively, and the detection rate per core for combined targeted biopsy (14.7%) was higher than the detection rate per core of system biopsy (6.5%, p <0.001) (107). In a retrospective study of 135 patients, MRI combined with 3D TRUS targeted needle biopsy was performed before RP, and the SE of the detection of prostate index tumors was 95% (108). Siddiqui MM et al. compared 1003 patients with MRI/US combined with prostate targeted biopsy and standard biopsy. The results showed that the accuracy of targeted biopsy was 30% higher than that of standard biopsy in diagnosing high-risk cancers (109). Tewes S et al. reported the SE, SP, and NPV of MRI/TRUS combined-guided targeted biopsy in detecting prostate lesions with PI-RADS scores ≥ 4 were 85%, 82% and 92%, respectively (110). US and MRI have advantages in the diagnosis of PCa, and when combined, the detection of PCa is obviously improved.

## mpUS

**Figure 2:**
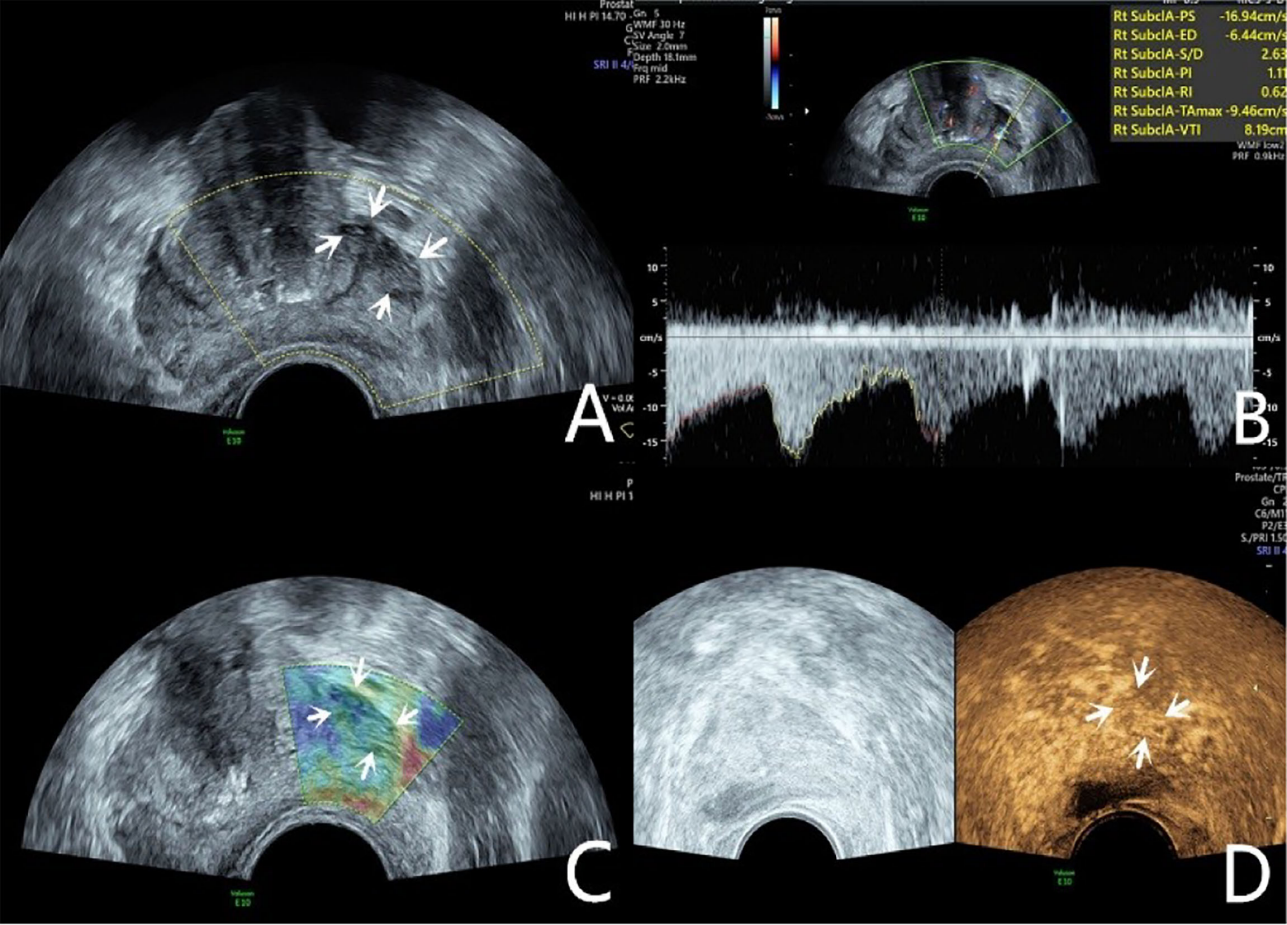
Diagnosis of PCa using mpUS—case 2. A 76-year-old patient has a total PSA of 14.3ng/ml. Multiparameter US starts from conventional transrectal ultrasound, and the lesion is a hypoechoic nodule at the junction of the inner and outer glands in the prostate’s left lobe (**A**, arrow). The lesion appears on the CDU as a rich blood flow of the arterial spectrum (**B**, arrow). The operator uses the endocavitary transducer to alternately compress and decompress the lesion, which appears mostly blue on the TRTE (**C**, arrow). Hypoechoic nodule appears on CEUS as hypervascular nodule with enhanced “fast forward and fast exit” (**D**, arrow). Histopathology shows that the prostate lesions were clinically significant with a Gleason 4 + 3 PCa.

Transrectal multimodal ultrasound refers to a combination of GSU, CDU, PDU, TRTE, and CEUS. GSU shows the anatomical location of the prostate lesion (**Figure 2A**). Doppler ultrasound shows the blood flow in the larger hyperplastic vessels in the lesion (**Figure 2B**). Elastography shows the hardness of the lesion tissue to infer properties about its nature (**Figure 2C**). CEUS shows new microvessels in the lesion (**Figure 2D**). Clinically, the combination of different ultrasound modes can improve the detection rate of PCa. At present, there are few studies on the combination of ultrasound modes.

Nelson et al. used GSU, PDU, and TRTE for the targeted biopsy of prostate lesions in 137 patients. The results showed that in 106 positive sextant sites, the positive rates of GSU, CDU, TRTE, and combined ultrasound modes were 16%, 29%, 25%, and 46%, respectively, indicating that combined ultrasound methods with different modes can improve the detection rate of PCa (17). Xie et al. conducted transrectal GSU, PDU and their combination with a third mode (DCE-US) to detect PCa in 150 patients. The results showed that the sensitivities of the combined mode (GSU+PDU), GSU, PDU and DCE-US, were 73%, 51%, 48%, and 63%, respectively (111). In a retrospective study involving 133 men with elevated serum levels of PSA (≥1. 25 ng/mL), the PCa detection rate of CEUS and TRTE combined targeted biopsy was 59.4% (112). Brock et al. performed TRTE and CEUS examinations on 100 patients before RP. Compared with pathological biopsy, the examinations showed a SE and SP in detecting PCa by TRTE of 49% and 74%, respectively. Compared with the combination of TRTE and CEUS, the false positive value of TRTE alone was reduced from 34.9% to 10.3%, and the PPV of cancer detection was increased from 65% to 90% (64). Among 153 prostate nodules, the SE, accuracy and NPV of the combination of TRTE and CEUS in diagnosing PCa were 92.1%, 86.2%, and 84.6%, respectively. Multiple ultrasound imaging modes combined with targeted-guided prostate biopsy can not only increase the detection rate of malignant lesions but also reduce the number of tissue punctures.

Mp-US and mp-MRI provide complementary information in the diagnosis of PCa. Zhang et al. performed mp-US and mp-MRI examinations on 88 patients. The results showed that the SE, NPV, accuracy, and AUC in detecting PCa with mp-US were higher than that mp-MRI (97.4% vs. 94.7%, 96.9% vs. 92.3%, 87.2% vs. 76.9%, 0.874 vs. 0.774, respectively) (113). In 167 patients with primary prostate biopsy, Pat F. Fulgham et al. found that mpUS-targeted biopsy was superior to mpMRI/TRUS fusion-targeted biopsy in terms of the positive rate of PCa and the ability to detect low-malignancy PCa (114). Mp-US has higher diagnostic performance than mp-MRI in diagnosing local PCa.

## Conclusion

Due to the poor prognosis of metastatic PCa, early detection of PCa is the most effective strategy to reduce morbidity and mortality. MRI experts from the European Society of Urogenital Radiology (ESUR) developed the PI-RADS scoring system for prostate mpMRI and used Likert-type scales to score the corresponding lesions. At present, mpMRI is still used as the main imaging method for diagnosing PCa in clinical practice, and no multiparameter ultrasound image scoring system has been developed. Ultrasound is also very important in the imaging diagnosis of PCa, due to its low cost, ease of use, real-time functionality, lack of radiation, and the continuing development of more advanced ultrasound techniques. Polymeric NBs targeting PSMA as a new UCA can increase the diagnostic potential of CEUS and may become a popular research topic for targeted ultrasound molecular imaging of PCa. In addition, NBs can be used as drug carriers for PCa-targeted therapy. Ultrasound molecular imaging has become an emerging research field in tumor imaging diagnosis. Our future work will focus on accumulating more patient data, integrating the diagnostic characteristics of PCa under different ultrasound modes, and constructing a complete ultrasound examination scoring system through optimized algorithms. The development of this advanced mpUS scoring system will have important clinical application value in improving PCa diagnosis and follow-up.

## Author Contributions

YL analyzed relevant literatures and drafted the article. RX made a critical review of the intellectual content of the article. SZ provided project funding. All authors read and approved the final manuscript.

## Funding

State Natural Sciences Foundation of China,Grant/Award Numbers: 81871372. The project leader is SZ.

## Conflict of Interest

The authors declare that the research was conducted in the absence of any commercial or financial relationships that could be construed as a potential conflict of interest.

## Publisher’s Note

All claims expressed in this article are solely those of the authors and do not necessarily represent those of their affiliated organizations, or those of the publisher, the editors and the reviewers. Any product that may be evaluated in this article, or claim that may be made by its manufacturer, is not guaranteed or endorsed by the publisher.

## Glossary

**Table:**

PCa	Prostate cancer
DRE	Digital rectal examination
PSA	Prostate specific antigen
TRUS	Transrectal ultrasound
SE	Sensitivity
SP	Specificity
MRI	Magnetic resonance imaging
US	Ultrasound
mp-MRI	Multiparameter MRI
T2WI	T2- weighted imaging
DWI	Diffusion weighted imaging
DCE-MRI	Dynamic contrast-enhanced MRI
mp-US	multiparametric ultrasound
EAU	European Urology Association
3D-TUS	Transrectal three-dimensional ultrasound
DOR	Diagnostic odds ratio
AUC	Area under curve
csPCa	Clinically significant prostate cancer
ANNA/C-TRUS	Artificial neural network analysis/computerized-transrectal ultrasound
RP	Radical prostatectomy
NPV	Negative predictive value
PPV	Positive predictive value
HS	Histoscanning
PHS-TT	Prostate tissue scanning targeting
TTB	Transperineal template prostate biopsy
GSU	Grayscale ultrasound
CDU	Color Doppler ultrasound
PDU	Power Doppler ultrasound
MVD	Microvessel density
UE	Ultrasound elastography
TRTE	Transrectal real-time tissue elastography
SWE	Shear-wave elastography
ROI	Region of interest
RBIE	Real-time balloon inflatable elastography
SD	Strain index
ARFI	Acoustic radiation force pulse imaging
CEUS	Contrast-enhanced ultrasound
UCA	Ultrasound contrast agent
MBs	Microbubbles
TIC	Time intensity curve
3DCE-PDU	Three-dimensional contrast-enhanced Doppler ultrasound
CUDI	Contrast-enhanced ultrasound diffusion imaging
EIR	Early irrigation rate
MTT	Mean transit time
RT	Rise time
WIT	Wash-in time
PSMA	Prostate-specific membrane antigen
NBs	Nanobubbles
ICG	Indocyanine green
scFv	Single-chain variable fragment
ESUR	European Society of Urogenital Radiology
